# [Retracted] Cognitive improvements and reduction in amyloid plaque deposition by saikosaponin D treatment in a murine model of Alzheimer’s disease

**DOI:** 10.3892/etm.2024.12676

**Published:** 2024-08-05

**Authors:** Li Zhou, Jin-Yuan Huang, Di Zhang, Ya-Liang Zhao

Exp Ther Med 20:1082–1090, 2020; DOI: 10.3892/etm.2020.8760

Following the publication of this paper, it was drawn to the Editors’ attention by a concerned reader that the hemotoxylin and eosin-stained images shown in Fig. 2A, the immunofluorescence images shown in Fig. 2B, the TUNEL immunofluorescence images shown in Fig. 2D and the immunohistochemical images shown in Fig. 4A and C were strikingly similar to data appearing in different form in other articles written by different authors at different research institutes that had already been published elsewhere prior to the submission of this paper to *Experimental and Therapeutic Medicine*. In view of the fact that the abovementioned data had already apparently been published previously, the Editor of *Experimental and Therapeutic Medicine* has decided that this paper should be retracted from the Journal. The authors were asked for an explanation to account for these concerns, but the Editorial Office did not receive a reply. The Editor apologizes to the readership for any inconvenience caused.

